# Causal relationship between body mass index, type 2 diabetes and bone mineral density: Mendelian randomization

**DOI:** 10.1371/journal.pone.0290530

**Published:** 2023-10-02

**Authors:** Weiwei Ma, Xiaohong Zhou, Xin Huang, Yong Xiong

**Affiliations:** _School of Acupuncture-Moxibustion and Orthopedics, Hubei University of Chinese Medicine, Wuhan, 430061, China_; University of Calcutta, INDIA

## Abstract

**Objective:**

To reveal the relationship between Body Mass Index(BMI), type 2 diabetes, and bone mineral density(BMD) using a mendelian randomization (MR) approach.

**Methods:**

GWAS data on BMI, type 2 diabetes, and BMD were selected from the IEU GWAS database at the University of Bristol.Univariable, multivariable, and mediated MR analyses were used to explore the relationship between BMI, type 2 diabetes, and BMD. beta(β) values were given, and three methods, including inverse variance weighting, MR-Egger regression, and weighted median, were used in this analysis.

**Results:**

Univariable mendelian randomization (UVMR) results showed that BMI and type 2 diabetes were positively associated with BMD. However, the association between BMI and BMD was insignificant in the multivariable Mendelian randomization (MVMR) analysis, while that between type 2 diabetes and BMD remained significant. Mediated MR analysis indicated that type 2 diabetes mediated the regulation of BMD by BMI.

**Conclusion:**

This study provides evidence supporting a positive causal association between BMI, type 2 diabetes, and BMD. Type 2 diabetes acts as a mediator in the regulation of BMD by BMI, indicating that both BMI and type 2 diabetes exert a protective influence on BMD.

## 1 Introduction

Osteoporosis (OP) is a systemic bone metabolic disorder characterized by reduced bone mass, gradual loss of bone trabeculae, and decreased bone mineral density (BMD) [1]. With the progression of society and changes in human lifestyles and dietary patterns, the prevalence of overweight and obesity has risen. The World Health Organization (WHO) defines overweight and obesity as the excessive accumulation of fat that has detrimental effects on human health and recommends the utilization of body mass index (BMI) as a diagnostic tool [2]. Some studies [3] have proposed a protective effect of higher BMI against OP, with positive correlations observed between BMI values and BMD. However, the development of type 2 diabetes is closely associated with BMI, and research has demonstrated that an increase in BMI raises the risk of type 2 diabetes onset [4–9]. Moreover, the correlation between BMD and BMI can be bidirectional [10], being positive in cases of relative obesity (BMI 18. 0–31. 2 kg/m^2^) and negative in severe obesity scenarios (BMI 31. 3–40. 6 kg/m^2^). These findings indicate that conventional observational studies investigating the association between type 2 diabetes, BMI, and BMD may be influenced by potential confounding factors and reverse causality, potentially leading to biases and inaccurate conclusions.

Mendelian randomization (MR) employs genetic variation as an instrumental variable to establish causal associations between risk factors and disease. This method effectively addresses the issues of potential confounding and reverse causality, making it a valuable complement to traditional epidemiological methods [11]. multivariable Mendelian randomization (MVMR) is an extension of Univariable mendelian randomization (UVMR) that takes into account polymorphism of multiple traits [12]. The assumptions of MVMR are more inclusive, as genetic variation may impact several measured exposures, and the exclusion restrictions and exchangeability assumptions are accordingly expanded. MVMR gives consistent results in estimating the direct effect of primary exposure on the outcome, without the confounding effects of secondary exposures acting as mediators.

The study utilized UVMR and MVMR to investigate the effects of type 2 diabetes and BMI on BMD. Sensitivity analyses were conducted to assess the impact of various hypotheses on the study results and to ensure the robustness. A mediated MR analysis was performed to assess whether the effect of BMI on BMD was mediated by type 2 diabetes.

## 2 Materials and methods

All included studies were permitted by their academic ethics review committees, and each participant signed written informed consent. Ethical approval and consent to participate in the original GWASs were obtained from relevant review boards. This study was a re-analysis based on publicly available GWAS data; hence, no additional ethical approval was required.

### 2.1 Sources of information

The data used in this study were obtained from the IEU GWAS database at the University of Bristol (https://gwasmrcieu.ac.uk). Summary-level data for BMI were obtained from a 2018 meta-analysis of GWASs of height [13]. This meta-analysis uses a fixed-effects model that combined results from a GWAS of BMI conducted on 456,426 participants from the UK Biobank (adjusted for age, sex, recruitment center, genotyping batch, and 10 genetic principal components), with results from a 2014 GWAS published by the Genetic Investigation of ANthropometric Traits (GIANT) consortium. The GIANT GWAS had 253,288 participants from 79 studies (adjusted for age, height, sex, and study-specific covariates) [13]. Summary data on the associations of genetic variants with clinician-diagnosed type 2 diabetes were obtained from a recent GWAS meta-analysis of 62,892 type 2 diabetes patients and 596,424 controls of European ancestry, with 16 million gene variations [14]. The study included three contributing studies, namely the UK Biobank (UKB) full cohort release, Genetic Epidemiology Research on Aging (GERA), and Diabetes Genetics Replication and Meta-analysis (DIAGRAM) [14]. The BMD GWAS summary dataset included 56,284 individuals of European ancestry, and more information is available in the original study [15]. Using linear regression models, the SNPs associated with BMD were adjusted for covariates such as age, weight, height, etc. [15]. Detailed information is given in **Table 1**.

**Table 1:** Summary information on the data from the genome-wide association studies used in the MR analysis.

Variables	Trait	Sample size	Number of SNPs	Population	Year
BMI	body mass index	681,275	2,336,260	European	2018
Type 2 diabetes	Type 2 diabetes	655,666	5,030,727	European	2018
BMD	Total body bone mineral density	56284	16,162,733	European	2018

### 2.2 Univariate Mendelian randomization

**Fig 1:**
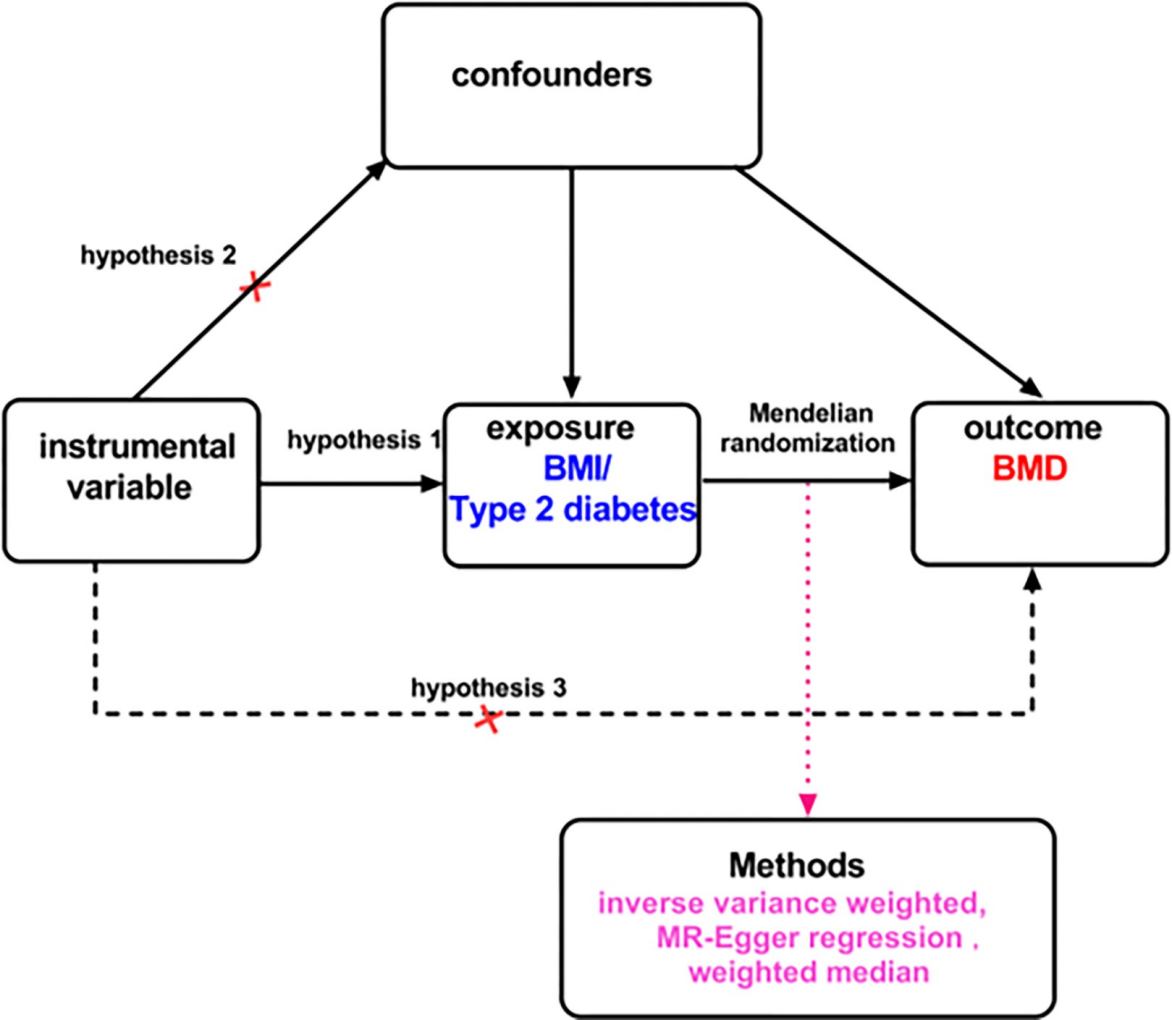
Overview of the study design and assumptions of the UVMR design. The UVMR analysis in this study satisfies the following three hypotheses: (1)there is a strong association between instrumental variables and exposure factors; (2) no confounding factors exist in the association between exposure and outcome, in other words, there is no genetic pleiotropy; and (3) the instrumental variables do not have a direct effect on the outcome and only influence the outcome through the exposure factor.

We conducted separate MR studies to investigate the causal relationship between BMI with type 2 diabetes (the exposure) and BMD (the outcome) utilizing GWAS data. **Fig 1** provides an overview of the study design and assumptions of the MR study. The instrumental variables (IVs) for the exposure traits were selected according to several criteria in the univariable MR analyses [16]. Specifically, the IVs should be strongly associated with exposure traits (*P* < 5×10^−8^), independent of each other as quantified by linkage disequilibrium (LD) of R^2 ^< 0.001, which was achieved by clumping with a 10 Mb window. Moreover, the IVs should have at least 10 variants, and the single nucleotide polymorphisms (SNPs) should be biallelic.

The main two-sample MR method used in this study was inverse variance weighting (IVW) [17], followed by MR-Egger [18] and weighted median [19]. Compared with IVW, the standard error of the causal estimate from the MR-Egger method is typically large, resulting in low causal estimates [20]. The MR-Egger method was used to investigate the potential bias introduced by pleiotropy and also provides an intercept test to determine whether an unbiased estimate of the causal effect exists [21]. The weighted median analysis calculates the median of an empirical distribution of MR association estimates, weighted for their precision. It provides consistent estimates when more than half of the instruments are valid [22]. If all included SNPs satisfy the assumption of being a valid tool variable, IVW could provide accurate estimates [23]. Hence, IVW is considered the main result when no weak IVs exist. When no more than 50% of the weight in the analysis is accounted for by the effective IVs, the weighted median method could offer a plausible estimate of the causal relationship [24]. To assess horizontal multiplicity in the MR analysis, the study conducted the MR-Egger intercept test. If the intercept term in this analysis was significant, it indicated the presence of horizontal multiplicity [25]. Additionally, Cochran’s Q statistic was used to detect heterogeneity, and a significant result indicated significant heterogeneity in the analysis [26].

### 2.3 Multivariable Mendelian randomization

**Fig 2:**
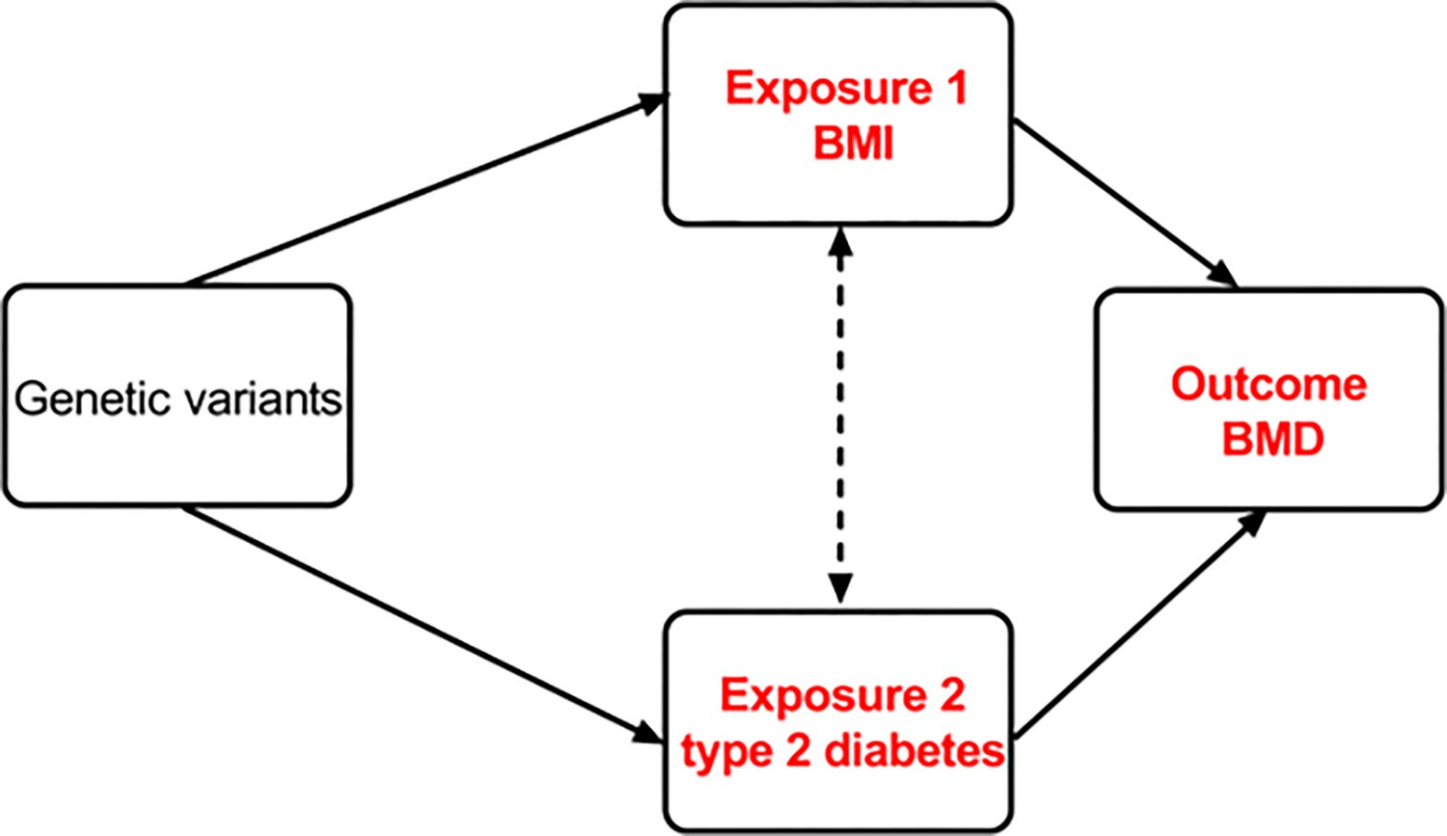
MVMR experimental design.

To account for potential confounding or mediating effects, we conducted an MVMR analysis, which allows for identifying causal effects of multiple risk factors, so that the direct effects of BMI and type 2 diabetes on BMD [27] can be revealed. The MVMR experimental design is shown in **Fig 2**. The MVMR technique accounts for the interrelationship between BMI and type 2 diabetes, and the IVs employed in the mvMR analysis are frequently linked to all exposures. Combinations of IVs from each exposure made up the SNPs utilized to conduct multivariable MR. SNPs associated with any BMI or type 2 diabetes were merged by removing duplicates with higher p-values. Relevant data from the original exposure datasets were extracted for these SNPs, which were then used as IVs in the MVMR analysis. The SNPs that were significantly (P threshold <  5 × 10^−8^) associated with BMI and type 2 diabetes were selected as instrumental variables, respectively. Independent variants (r^2^ < 0.001, window size  =  10,000 kb) were retained according to European ancestry reference data from the 1000 Genomes Project [16].

For multivariable MR analysis, we utilized IVW [17] with multiplicative random effects as the main analysis and MR-Egger [18] with multiplicative random effects methods as the complementary analysis to appraise the causal effects of BMI and type 2 diabetes on BMD. To evaluate the strength of the instruments used, we calculated F-statistics, where an F value greater than 10 indicates that the SNPs can effectively predict the exposures. When the F-statistic is less than 10, the genetic variation used is considered a weak instrumental variable, which may introduce bias into the results, and caution is required when interpreting the results [28,29]. In order to ensure the robustness of the instrumental variables, we initially computed the R^2^ value, which elucidates the extent of phenotypic variation expounded by all SNPs in the analysis. Subsequently, the instrumental strength of the SNPs for each socioeconomic trait was evaluated through the utilization of the F-statistic. An F-statistic exceeding 10 signifies that the composite SNP serves as a highly potent instrument for elucidating phenotypic variation, while an F-statistic equal to or less than 10 indicates a weak instrument.

### 2.4 Mediated Mendelian randomization

**Fig 3:**
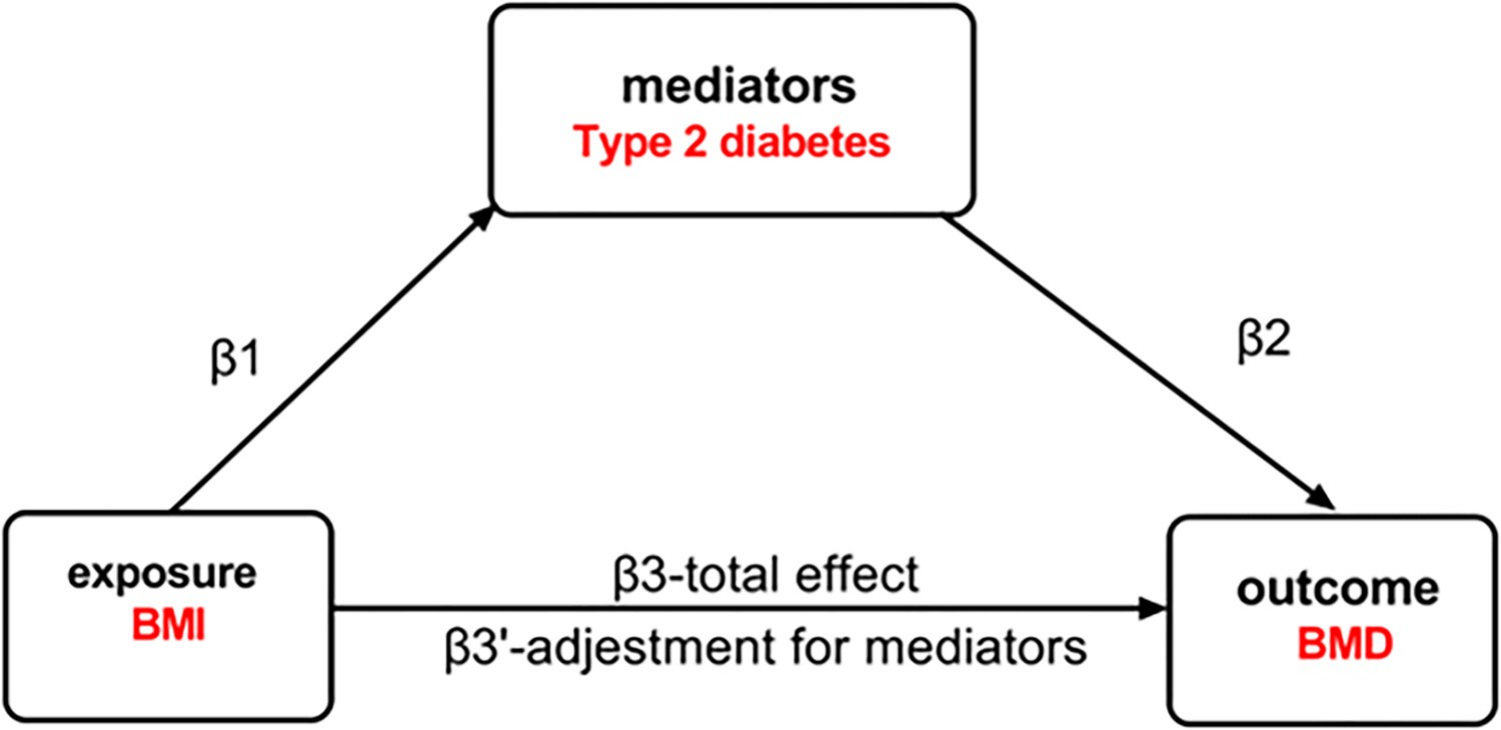
Graph of the proposed mediation by mediators for the association of BMI with BMD. β1 represents the regression coefficients for the association between BMI and mediators, β2 represents the regression coefficients for the association between mediators and BMD, and β3 represents the total effect between BMI and BMD without adjustment for mediators. Additionally, β3’ represents the direct effect between BMI and BMD, considering adjustment for mediators.

To explore the potential mediating role of type 2 diabetes in the association between BMI and BMD, we employed two-step MR and MVMR approaches, as illustrated in **Fig 3**. The two-step approach is considered less prone to biases inherent in the common multivariable approach [30]. In MVMR, the total effect of each exposure is decomposed into direct and indirect effects. A graphical representation of the analyses is depicted in Fig 2. Mediation was considered present if the following conditions were met: 1) a correlation existed between BMI and mediators (β1); 2) BMI was associated with BMD without adjusting for mediators (β3); 3) mediators were associated with BMD (β2). The mediation ratio was calculated as (β1×β2)/(β3), with an indirect effect of β1 ×β2 and a total effect of β3 +β1 ×β2.

### 2.5 Statistical analysis

Our study used the “TwoSampleMR” [31] and “Mendelian Randomisation” [32] packages in the R Studio software for exposure and outcome analysis. MR results are expressed as beta(β), interpreted as the effect of BMI and type 2 diabetes on BMD. We also report the corresponding lower and upper 95% confidence intervals (CIs) for all causal estimates. P-values <0.05 were used to define statistical significance.

## 3 Results

### 3.1 Univariate Mendelian randomization

**Fig 4:**
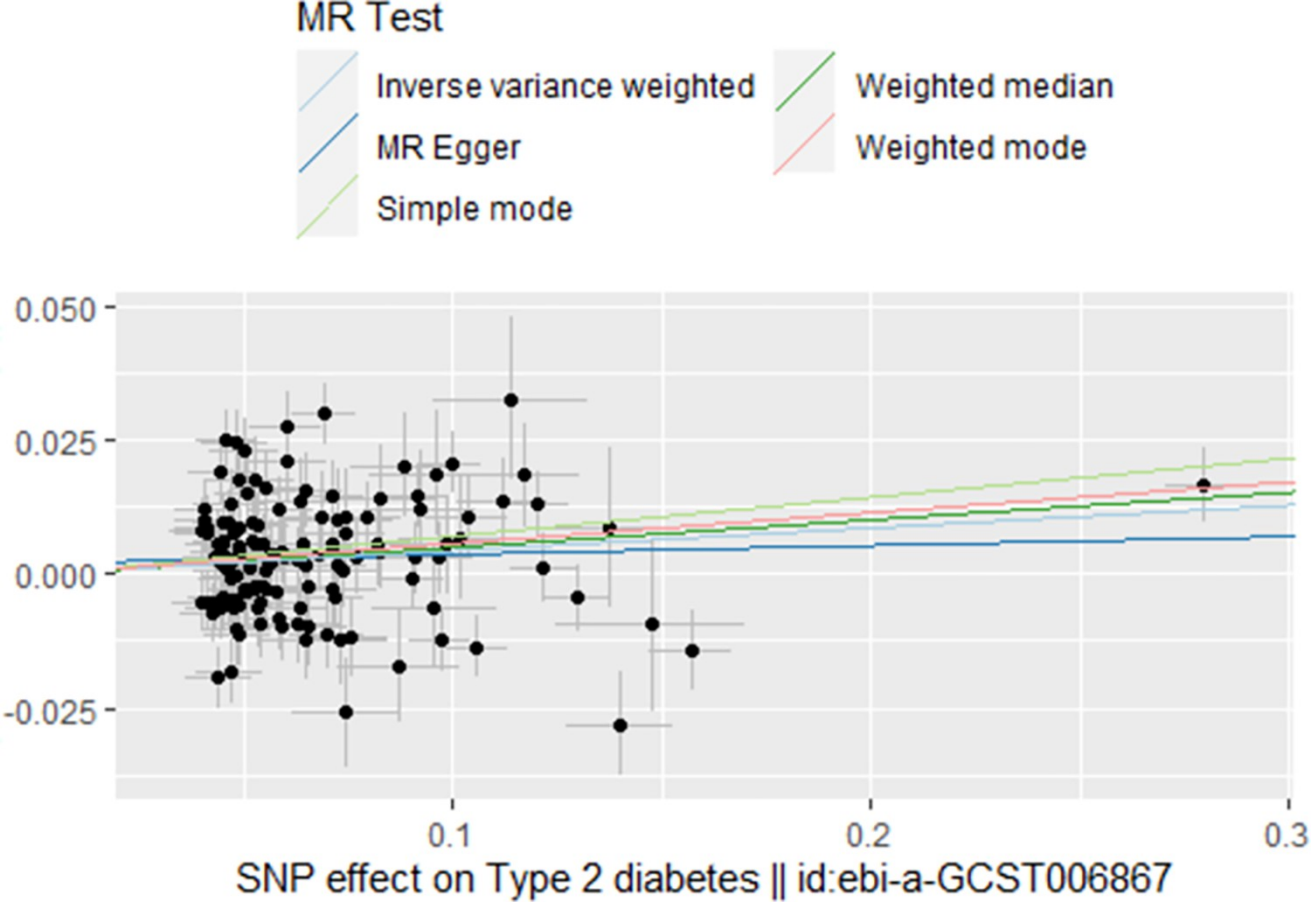
Scatter plot to visualize the causal effect of type 2 diabetes on BMD.

When considering type 2 diabetes as the exposure and BMD as the outcome, both the IVW and weighted median analyses indicated a protective effect of type 2 diabetes on BMD, with β of 0.04 (95% CI 1.01–1.06; *P* = 0.0008) and 0.05 (95% CI 1.02–1.08; *P* = 0.0007), respectively. However, the MR-Egger regression did not show a significant relationship between type 2 diabetes and BMD (β = 0.01, 95% CI 0.95–1.08; *P* = 0.59), as shown in **Table 2**. The direction of the causal effect was the same for all three methods (**Fig 4**). Although the MR-Egger regression results showed no horizontal pleiotropy between all genetic variants (intercept 0.0019; 0.37), there was evidence of heterogeneity in either IVW analysis (Q = 329.7, *P* = 1.086296e-18) or MR-Egger analysis (Q = 327.5, *P* = 1.248428e-18), which led us to adopt a random effects model for our analysis. The absence of horizontal pleiotropy in the analysis suggests that the IVW analysis results should be considered the primary criterion for causality. Thus, it can be concluded that type 2 diabetes is a protective factor for BMD.

**Table 2:** Association between BMI/type 2 diabetes and bone mineral density risk under different methods.

Exposure	Outcome	Method	β	95% Cl	*P* value	F-statistics	R^2^ (%)
BMI	BMD	IVW(random effects)	0.01	1.01–1.09	0.01		
		Weighted median	0.04	0.99–1.09	0.08	46.74	0.375
		MR-Egger	0.15	1.02–1.29	0.01		
type 2 diabetes	BMD	IVW(random effects)	0.05	0.95–1.08	0.0008		
		Weighted median	0.04	1.02–1.08	0.0008	55.26	0.216
		MR-Egger	0.01	0.95–1.08	0.59		

**Fig 5:**
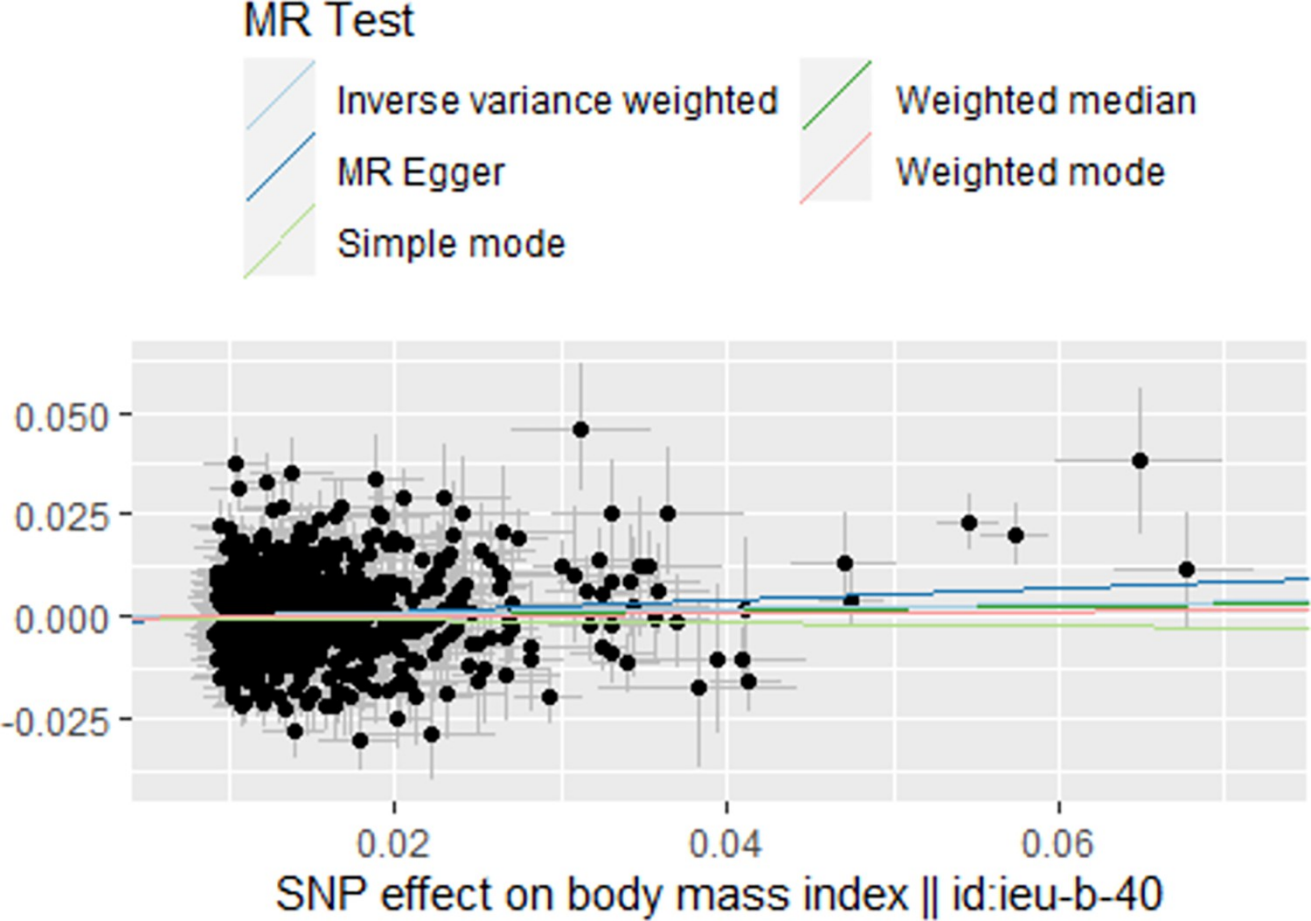
Scatter plot to visualize the causal effect of BMI on BMD.

Similarly, when considering BMI as the exposure and BMD as the outcome, the IVW and MR-Egger analyses also indicated a protective effect of BMI on BMD, with β of 0.05 (95% CI 1.01–1.09; *P* = 0.01) and 0.15 (95% CI 1.02–1.29; *P* = 0.01), respectively. However, the weighted median analysis did not show a significant relationship between BMI and BMD (β = 0.04, 95% CI 0.99–1.09; *P* = 0.08), as shown in **Table 2**. The direction of the causal effect was the same for all three methods (**Fig 5**). Moreover, the MR-Egger regression results showed no horizontal pleiotropy between all genetic variants (intercept -0.001; 0.09), but there was evidence of heterogeneity in either IVW analysis (Q = 1701, *P* = 1.758784e-45) or MR-Egger analysis (Q = 1696, *P* = 4.014075e-45). Therefore, we also used the findings of the IVW analysis as the primary criterion for causality, concluding that BMI is a protective factor for BMD.

### 3.2 Multivariable Mendelian Randomization

To control for pleiotropic pathways that could confound the association between BMI and type 2 diabetes, we employed an MVMR model in which the combined effect of BMI and type 2 diabetes was treated as the exposure about BMD outcomes. The results from the IVW analysis indicated that the previously observed significant association between genetically predicted BMI and BMD by UNMR was attenuated in the MVMR model and was no longer statistically significant (IVW: β = -0.01, 95% CI 0.94–1.05, *P* = 0.93). The MR-Egger regression yielded similar results (β = 0.03, 95% CI 0.95–1.09, *P* = 0.51). In contrast, the protective effect of type 2 diabetes on BMD remained significant even after adjusting for BMI (IVW: β = 0.02, 95% CI 1.00–1.05, *P* = 0.02; MR-Egger:β = 0.01, 95% CI 1.008–1.071, *P* = 0.01). The F-statistic values in the MVMR were all greater than 10, indicating a low likelihood of bias in the results and high reliability and stability of the study findings. Detailed results are presented in **Table 3**.

**Table 3:** Results of multivariate Mendelian randomisation analysis.

Exposure	Outcome	Method	β	95% Cl	*P* value	F‐statistics
BMI	BMD	IVW	-0.01	0.94–1.05	0.93	71.73
		MR-Egger	0.03	0.95–1.09	0.51	
type 2 diabetes		IVW	0.02	1–1.05	0.02	67.17
		MR-Egger	0.01	1.008–1.071	0.01	

### 3.3 Mediated Mendelian randomization

We conducted a mediated MR analysis to examine the potential mediating role of type 2 diabetes in the association between BMI and BMD. The results are presented in detail in **Table 4**. Since there was no direct effect between BMI and BMD, the mediating effect was calculated as the indirect effect (β = β1 × β2). Our study found that type 2 diabetes was a mediator in the relationship between BMI and BMD, with a mediation effect estimate of β = 0.04 (OR = 1.04, 95% CI 1.02–1.06, *p* = 0.04).

**Table 4:** Results of intermediate Mendelian randomisation analysis.

Exposure	Mediated	Outcome	β	OR	95% Cl	*P* value
BMI		type 2 diabetes	0.98	2.67	2.36–3.03	2.922086e-53
type 2 diabetes		BMD	0.03	1.03	1.01–1.06	0.001
BMI		BMD	0.04	1.04	0.99–1.09	0.1
BMI	type 2 diabetes	BMD	0.04	1.04	1.02–1.06	0.04

## 4 Discussion

This study employed aggregated data from extensive GWAS to explore the interplay between BMI, type 2 diabetes, and BMD using MR analysis. In the two-sample MR analysis, both BMI and type 2 diabetes exhibited an elevated risk of OP. However, upon adjusting for type 2 diabetes, we observed a reduction in OP risk associated with BMI. Furthermore, mediated MR analysis unveiled that BMI contributed to an increased risk of OP, yet this effect was mediated by type 2 diabetes.

Numerous previous observational clinical studies have reported the association between BMI and BMD, although the impact of higher BMI on bone health remains a topic of debate. Some studies have demonstrated a positive correlation between BMI and BMD [33], with certain researchers proposing that maintaining adequate fat mass enhances BMD, particularly in postmenopausal women [34]. However, others have observed a negative correlation between fat mass and BMD, as increased fat mass can lead to heightened levels of pro-inflammatory cytokines, thereby accelerating bone resorption and loss [35]. Moreover, a prospective study identified obesity as a potential risk factor for fractures in postmenopausal women [36]. In our present study, we employed separate two-sample MR analyses and identified a causal association between BMI, type 2 diabetes, and OP. However, upon correcting the analysis using multisample MR, no causal association between BMI and OP was observed. This suggests the potential influence of confounding factors on the relationship between BMI and the development of OP, thereby introducing bias to the results. Given the intricate interplay of exposure factors in the clinical setting, joint modeling was employed to account for these interactions in the analysis.

Type 2 diabetes is confounding when analyzing the relationship between BMI and BMD. Some researchers employing MR analysis [37] provide support for a causal connection between BMI and type 2 diabetes, while multiple clinical studies [38–40] demonstrate a strong correlation. The utilization of MVMR to mitigate bias induced by type 2 diabetes revealed no causal association between BMI and OP, indicating that type 2 diabetes might serve as a potential mediator in the relationship between BMI and OP risk. These findings imply that the impact of BMI and type 2 diabetes on BMD entails a complex process. To further investigate the causal pathways, mediated MR models incorporating genetic tools to explore mediators are being contemplated, as they hold the potential to provide novel insights into causal relationships.

Previous studies have proposed several mechanisms for the association between BMI and BMD. One explanation is that an increase in BMI can promote mechanical stress on bone density, stimulating the proliferation, differentiation, and mineralisation of osteoblasts, effectively increasing bone density [41]. Additionally, skeletal muscle and bone are closely linked, and the stresses generated during muscle movement can directly affect bone. While bones will produce specific changes in order to adapt to the stress exerted by muscle. For example, synthesis and expression of osteoblast-related genes are increased; and the proliferation, differentiation and mineralisation of human osteoblasts and osteocytes will be significantly accelerated, ultimately increasing bone strength to adapt to stress changes [42]. Adiposity, as an endocrine marker, can secrete growth factors such as adiponectin and leptin to promote bone growth [43]. Furthermore, adipocytes can produce estrogen, which impacts bone metabolism, especially in postmenopausal women [44]. In addition to these mechanisms, our study revealed a potential mediation of type 2 diabetes in the relationship between BMI and BMD. Regarding the specific mechanisms involved, a multitude of factors are deemed accountable. Firstly, insulin, a hormone recognized for its regulatory function in bone anabolism, is believed to play a pivotal role in the pathogenesis of type 2 diabetes. Consequently, insulin levels may elucidate the elevated BMD levels observed in individuals with type 2 diabetes [45,46]. Insulin can directly impact osteoblast and osteoclast differentiation through insulin receptors, or indirectly influence them by regulating vitamin D and parathyroid hormone levels [47–49], thus exerting an influence on BMD levels. Furthermore, individuals with type 2 diabetes often exhibit insulin resistance, which leads to increased blood glucose levels due to inadequate insulin secretion. Under conditions of insulin resistance, the body compensates by augmenting insulin secretion to counteract this response, resulting in elevated blood insulin levels. This insulin resistance, along with compensatory high insulin levels, can contribute to enhanced bone mineral density [50,51]. Secondly, contemporary research has demonstrated that elevated blood glucose levels in vivo affect osteoclast differentiation and hinder osteoclast-mediated bone matrix degradation, thereby leading to increased BMD [52]. Lastly, thiazides [53] and statins [54], commonly employed in the treatment of type 2 diabetes, have been shown to promote BMD augmentation. In conclusion, the influence of BMI on BMD is considerable, and type 2 diabetes may serve as a significant mediating factor in this process.

The present study exhibits several noteworthy strengths. It represents the first application of MR in investigating the association between BMI, type 2 diabetes, and BMD within a European population. In contrast to conventional observational research methods, MR techniques effectively mitigate inherent limitations, such as confounding factors and reverse causality, which can impact result accuracy [55]. In this study, we employed SNPs with genome-wide associations and independent inheritance but no LD as IVs to enhance the reliability of our findings.While MR serves as a powerful approach to establish causal relationships between exposures and outcomes using summary statistics, it is imperative to interpret our findings cautiously due to several limitations. Firstly, our investigation relied on data derived from two extensive GWAS, and subgroup analyses were not feasible due to the absence of specific demographic information and clinical records of the study participants. Secondly, the presence of an ethnic bias is plausible in our study since the subjects were of European descent. Therefore, extrapolating our conclusions to other racial populations without further investigation may not be appropriate. Moreover, additional research is required to validate our findings and incorporate them into clinical diagnostic procedures and treatment options. Lastly, as all the data analyzed were sourced from databases, potential sample overlap could introduce bias to the findings due to weak instruments.Furthermore, considering the established correlation between BMI and type 2 diabetes, we conducted a mediated MR analysis with BMI as the exposure, type 2 diabetes as the mediator, and BMD as the outcome. Our outcomes indicate that the relationship between BMI and BMD might be mediated by type 2 diabetes, thereby providing novel insights for clinical research. Ultimately, given the high prevalence of obesity, type 2 diabetes, and OP within the population, our findings possess significant implications for healthcare policies. By elucidating the causal relationships among these conditions, we can better equip ourselves to implement early prevention strategies and timely interventions.

In summary, our study provides evidence of a direct positive association between type 2 diabetes and BMD, and suggests that the impact of BMI on BMD may be mediated by type 2 diabetes. These findings indicate that type 2 diabetes could serve as a protective factor for BMD and should prompt clinicians to consider this potential association. Nevertheless, further clinical investigations are required to unravel the intricate causal relationship between BMI, type 2 diabetes, and BMD.

## Supporting information

S1 File SNPs of exposure and mediators used in MR analyses. (XLS)
